# A nomogram model to predict grade ≥2 acute radiation enteritis in older adult patients with cervical cancer

**DOI:** 10.3389/fpubh.2025.1614073

**Published:** 2025-07-07

**Authors:** Lin Zhu, Lijuan Wang, Yihan Wu, Ming Zhu, Xue Huang, Yan Ma, Dandan Xu, Sen Wang, Yuxing Yang, Xiaoting Xu

**Affiliations:** ^1^Department of Gynecology, Fourth People's Hospital of Changzhou, Changzhou, China; ^2^Clinical Oncology Laboratory, Fourth People's Hospital of Changzhou, Changzhou, China; ^3^Department of Radiation Oncology (Gynecologic Oncology), Fourth People's Hospital of Changzhou, Changzhou, China; ^4^Department of Radiation Oncology, The First Affiliated Hospital of Soochow University, Suzhou, China

**Keywords:** cervical cancer, radiation enteritis, nomogram, older adult patients, radiotherapy

## Abstract

**Introduction:**

Acute Radiation Enteritis (ARE) is a common complication of pelvic radiotherapy, with incidence rates exceeding 60% in older adult populations. Especially, grade ≥2 ARE can lead to treatment interruptions, malnutrition, and even septic shock, thereby impairing patients’ quality of life and survival outcomes. However, existing risk prediction models are predominantly developed based on younger populations or mixed cohorts, lacking sophisticated evaluation tools tailored to older adult patients.

**Methods:**

To establish a predictive nomogram for grade ≥2 ARE in older adult cervical cancer patients undergoing radiotherapy, a retrospective cohort study of 251 older adult cervical cancer patients who received pelvic radiotherapy between January 2018 and March 2024 was conducted. Independent risk factors identified through univariate and multivariate logistic regression were incorporated into a nomogram. The model performance was validated using receiver operating characteristic (ROC) curves, calibration plots, and decision curve analysis (DCA).

**Results:**

The incidence of grade ≥2 ARE in our cohort was 61.35%. Independent risk factors included age (OR = 1.881, 95%CI: 1.015–3.484), hypertension (OR = 4.577, 95%CI: 2.402–8.720), diabetes (OR = 5.503, 95%CI: 2.206–13.726), Dmean_R (OR = 1.309, 95%CI: 1.155–1.483), and lactate dehydrogenase-to-albumin ratio (LAR), (OR = 1.872, 95%CI: 1.381–2.538). The nomogram exhibited strong discriminative ability (0.825, 95% CI: 0.774–0.877), and excellent calibration (Hosmer–Lemeshow test, *p* = 0.744).

**Conclusion:**

This nomogram integrates both clinical and dosimetric parameters to enable precise risk stratification for grade ≥2 ARE in older adult cervical cancer patients, facilitating personalized prevention strategies and optimized treatment planning.

## 1 Introduction

Cervical cancer (CC) is one of the most prevalent gynecologic malignancies globally. Radiotherapy serves as a cornerstone treatment for high-risk or inoperable cervical cancer patients (1, 2). Although CC primarily affects women aged 35–55 years, about 20% of cases occur in older adult patients (≥60 years) (3). Due to age-related physiological decline and comorbidities, older adult patients exhibit distinct radiotherapy tolerance and therapeutic responses in comparison with younger cohorts (4). Acute radiation enteritis (ARE) is a frequent complication of pelvic radiotherapy, manifesting as abdominal pain, diarrhea, mucoid/bloody stools, and increased infection risk. Severe cases may lead to life-threatening intestinal dysfunction complications (e.g., hemorrhage, perforation), which need treatment interruptions or surgical interventions. Given the high incidence of cervical cancer in older adult patients and their unique vulnerability to treatment complications, particular attention must be paid to ARE.

Current evidence indicates that ARE pathogenesis involves multifactorial interactions between clinical factors (e.g., age, hypertension, diabetes, prior abdominal/pelvic surgery) (4–6), dosimetric parameters (e.g., radiation dose/volume metrics) (7), and systemic inflammation markers (8). Some studies have also shown that the gut microbiota modulates the initiation and progression of radiation enteritis (RE) (9). However, no single factor reliably predicts ARE occurrence. Given the complexity of RE, it is really challenging for a single risk factor to accurately predict its occurrence. Therefore, multifactorial predictive models are urgently needed to guide personalized risk stratification and intervention strategies in older adult CC patients.

Existing risk prediction models are predominantly developed based on younger populations or mixed cohorts, lacking sophisticated evaluation tools tailored to older adult patients. This study retrospectively analyzed independent influencing factors of grade ≥2 ARE in older adult CC patients undergoing pelvic radiotherapy. Based on identified risk factors, we developed a nomogram model to aid in personalized risk assessment and provide a basis for further targeted intervention strategies.

## 2 Materials and methods

### 2.1 Study design and patient enrollment

We conducted a retrospective cohort study of older adult cervical cancer patients (age ≥60 years) who received pelvic external irradiation at the Affiliated Changzhou Fourth People’s Hospital of Soochow University between January 2018 and March 2024. Inclusion Criteria: (1) histopathologically confirmed cervical cancer; (2) pelvic external irradiation as primary or adjuvant therapy; (3) no prior history of intestinal diseases or pelvic radiotherapy; (4) complete ARE documentation; (5) Karnofsky Performance Status (KPS) ≥ 70. Exclusion Criteria: (1) comorbid liver dysfunction or hematologic disorders; (2) incomplete medical records; (3) severe hearing/communication impairments. A total of 251 patients met the inclusion criteria and were included in the final analysis. All procedures were approved by the Ethics Committee of the Affiliated Changzhou Fourth People’s Hospital of Soochow University (No. 2024–019) and conducted in compliance with the Declaration of Helsinki. Patient consent was waived due to the retrospective nature of the study.

### 2.2 Data collection

**Figure 1:**
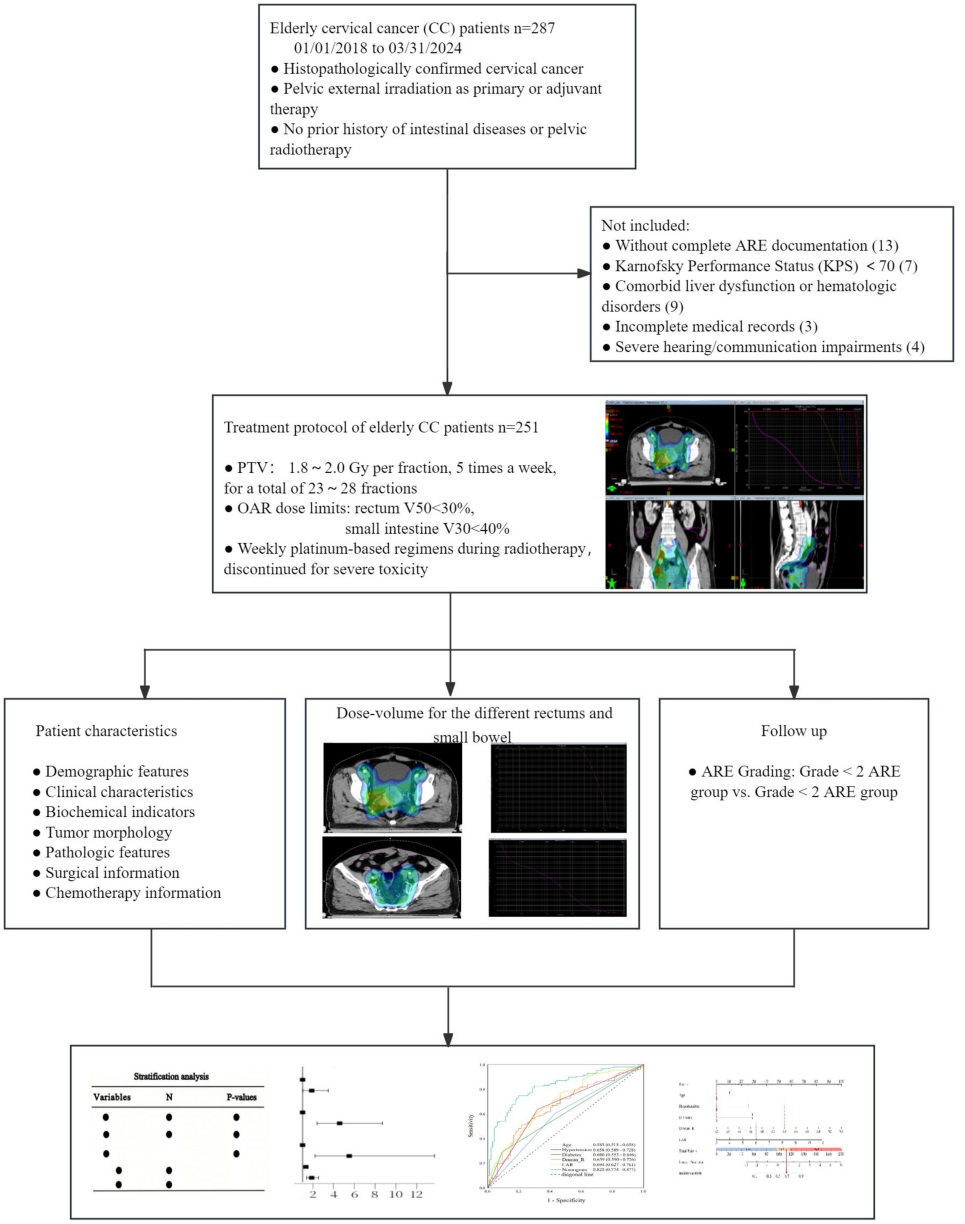
Study design and procedures.

A total of 287 older adult cervical cancer patients were collected, with 23 cases not meeting the eligibility criteria and 13 cases lacking ARE records. Finally, 251 cases were included. Clinical, hematological, and target area planning and dosage information of the patients were collected from the electronic medical record system, laboratory system, and Treatment Planning System (TPS) system. The flow chart is shown in Figure 1.

#### 2.2.1 Clinical parameters

Demographic and clinical data were collected, including: age, body mass index (BMI), hypertension, diabetes, FIGO staging (2018), vaginal invasion, surgical history, and concurrent/adjuvant chemotherapy.

#### 2.2.2 Dosimetric parameters

Statistics of dose-volume parameters (percentage of PTV volume receiving the prescribed dose) for the different rectums and small bowel, recorded as Vx. To illustrate, V50_R = 50% signifies that the volume of the rectum enclosed by the 50 Gy isodose line accounts for 50% of the total rectal volume. Similarly, V30_SI = 40% indicates that the volume of the small intestine enclosed by the 30 Gy isodose line represents 40% of the total small intestine volume. Dmax_R denotes the maximum dose received at any point in the rectum, while Dmax_SI is used to denote the maximum dose received at any point in the small intestine.

#### 2.2.3 Laboratory biomarkers

Blood samples were collected within 1 week pre-treatment to measure hematological data: including peripheral blood hemoglobin (HB, g/L), lactate dehydrogenase (LDH, U/L), albumin (ALB, g/L), C-reactive protein (CRP, mg/L), and the lactate dehydrogenase-to-albumin ratio (LAR). LAR = LDH/ALB.

#### 2.2.4 ARE grading

According to the Common Terminology Criteria for Adverse Events (CTCAE 5.0) from the National Institutes of Health: Grade 0: no change in bowel habits; Grade 1: increased frequency of bowel movements without the need for medical intervention; Grade 2: diarrhea up to 5 times a day or hematochezia without the need for sanitary pads, or rectal discomfort or abdominal pain; Grade 3: diarrhea more than 5 times a day or hematochezia requiring sanitary pads, or requiring parenteral nutritional support; Grade 4: acute or subacute intestinal obstruction, fistula or perforation, or massive hemorrhage requiring transfusion; Grade 5: death in the patient.

Follow-up was performed from the start of external irradiation until 3 months after the end of radiotherapy. Patients’ adverse reactions were assessed weekly, and more frequently if intervention was required. After the completion of external beam radiation, follow-up visits were scheduled on a monthly basis. During the follow-up period, all patients received a clinical RE grade, with the highest grade recorded as the final grade.

### 2.3 Treatment protocol

#### 2.3.1 External beam irradiation

Forty minutes before CT positioning, the patient was instructed to empty the bladder and then drink 800 mL of water to refill the bladder. The patient took the supine position and immobilized with a body membrane. The CT scanning area was the 10th thoracic vertebrae to 10 cm below the sciatic tuberosity (slice thickness: 5 mm). The CT localization images were transmitted to a treatment planning system (Eclipse, Varian, United States). Clinical target volume (CTV) included the cervix, vagina (3 cm below the margin of tumor), uterus and parametrium, invaded lymph nodes, and pelvic/para-abdominal aortic lymph node drainage areas. The planning target volume (PTV) was defined as a three-dimensional extrapolation of 0.5 cm from the clinical target volume (CTV), with the requisite modifications for anatomical barriers and neighboring organs at risk.

In this study, Varian Clinac IX linear accelerator (6MV X-ray, IMRT/VMAT) was used, with a prescription dose of PTV of 1.8 ~ 2.0 Gy per fraction, 5 times a week, for a total of 23 ~ 28 fractions. The total dose to the paraaortic lymphatic drainage area was 40 ~ 45 Gy, and the total dose to the pelvic area was 45.0 ~ 50.40 Gy positive lymph nodes 56 ~ 60.2 Gy. Dose requirements: ≥95% of PTV receives prescribed dose and maximum dose in PTV is <110% of prescribed dose. OAR dose limits: rectum V50 < 30%, small intestine V30 < 40%, bladder V50 < 30%, kidneys V15 ≤ 50%, spinal cord V45 < 5%. Patients received a total dose of 80 ~ 85 Gy for radical radiotherapy and 65 ~ 70 Gy for postoperative adjuvant radiotherapy, which includes both external beam radiation therapy (EBRT) and brachytherapy.

#### 2.3.2 Chemotherapy regimen

(1) Concurrent chemotherapy: weekly platinum-based regimens during radiotherapy. (2) Adjuvant chemotherapy: paclitaxel plus platinum every 21–28 days after the completion of radiotherapy (discontinued for severe toxicity).

### 2.4 Statistical analysis

SPSS 26.0 and R.4.2.3 were used for the statistical analyses. Continuous variables were assessed for normality, non-normally distributed continuous measures were expressed as median and interquartile spacing, and intergroup comparisons were made using the Mann–Whitney *U* test; count data were expressed as frequency (constitutive ratio), two-group comparisons were made using the chi-square test, and multi-group ranked count data were made using the Kruskal–Wallis rank-sum test. Risk factors for screening ≥ grade 2 ARE were analyzed using one-way logistic regression, and *p* < 0.05 was considered a statistically significant difference. Independent risk factors for grade ≥2 ARE according to multifactorial logistic regression analysis. Subgroup analysis was conducted based on age strata.

A nomogram was constructed using the independent risk factors in the multifactorial analysis. Internal validation was performed using the Bootstrap method (number of times = 1,000), the area under the ROC curve (AUC) was used to evaluate the differentiation of the model, the calibration curve was plotted to evaluate the accuracy of the model, and the clinical applicability of the model was evaluated using decision curve analysis (DCA). The optimal thresholds for age, hypertension, diabetes, Dmean_R dose, and LAR were calculated based on the subjects’ work characteristics (ROC) curves, respectively, in order to select the most relevant thresholds for the prediction of grade ≥2 ARE. *p* < 0.05 was taken as a statistically significant difference.

## 3 Results

### 3.1 Clinical data analysis

A total of 251 cervical cancer patients were enrolled in this study. Among them, 97 cases (38.65%) developed grade <2 ARE, while 154 cases (61.35%) experienced grade ≥2 ARE. Within the grade <2 ARE subgroup, 45 patients (17.93%) exhibited no ARE symptoms, and 52 patients (20.71%) presented with grade 1 ARE. In the grade ≥2 ARE subgroup, 139 patients (55.38%) were classified as grade 2, and 13 patients (5.18%) as grade 3 (all requiring temporary radiotherapy suspension followed by symptom management to complete treatment). Notably, 2 cases (0.79%) progressed to grade 4 ARE, including 1 intestinal perforation and 1 severe anemia requiring blood transfusion, both leading to treatment discontinuation. The clinical characteristics and biochemical indicators were presented in Table 1. Among these parameters, age, hypertension, diabetes, lymph node metastasis, FIGO staging, vaginal invasion, surgical intervention, albumin (ALB), lactate dehydrogenase (LDH), and lactate dehydrogenase to albumin ratio (LAR) demonstrated statistically significant associations with the incidence of grade ≥2 ARE (*p* < 0.05) (Table 1).

**Table 1:** Comparison of general data between the two groups.

Characteristics	Total cases (*n* = 251)	Grade < 2 ARE group (*n* = 97)	Grade ≥ 2 ARE group (*n* = 154)	*p*
Weight (Kg)	57.50 (53.00, 65.00)	57.00 (53.00, 64.00)	58.00 (54.00, 65.00)	0.296
Tumor size (cm)	4.00 (3.50, 5.00)	4.00 (3.50, 5.00)	4.00 (3.50, 5.00)	0.448
ALB (g/L)	39.60 (36.65, 42.00)	40.50 (37.90, 42.80)	39.50 (36.50, 41.60)	**0.014**
LDH (U/L)	183.00 (164.00, 18.00)	170.00 (154.00, 188.00)	188.00 (166.00, 229.75)	**<0.001**
LAR	4.61 (4.06, 5.55)	4.23 (3.85, 4.86)	4.83 (4.33, 5.91)	**<0.001**
HGB (g/L)	116.00 (100.00, 126.00)	116.00 (106.00, 128.00)	115.50 (98.00, 125.00)	0.123
CRP (mg/L)	1.99 (0.68, 6.26)	1.50 (0.60, 3.84)	2.34 (0.82, 6.71)	0.152
Age (y), *n*(%)				**0.009**
60–69	116 (46.22)	55 (56.70)	61 (39.61)	
≥70	135 (53.78)	42 (43.30)	93 (60.39)	
BMI, *n*(%)				0.605
<18.5	12 (4.78)	5 (5.15)	7 (4.55)	
18.5–23.9	136 (54.18)	56 (57.73)	80 (51.95)	
>23.9	103 (41.04)	36 (37.11)	67 (43.51)	
Hypertension, *n*(%)				**<0.001**
No	122 (48.61)	66 (68.04)	56 (36.36)	
Yes	129 (51.39)	31 (31.96)	98 (63.64)	
Diabetes, *n*(%)				**<0.001**
No	197 (78.49)	88 (90.72)	109 (70.78)	
Yes	54 (21.51)	9 (9.28)	45 (29.22)	
Histological type, *n*(%)				0.957
Squamous cell carcinoma	230 (91.63)	89 (91.75)	141 (91.56)	
Non-squamous cell carcinoma	21 (8.37)	8 (8.25)	13 (8.44)	
Degree of Differentiation, *n*(%)				0.884
High and medium differentiation	185 (73.71)	71 (73.20)	114 (74.03)	
Low differentiation	66 (26.29)	26 (26.80)	40 (25.97)	
Lymphatic metastasis, *n*(%)				**0.013**
No	191 (76.10)	82 (84.54)	109 (70.78)	
Yes	60 (23.90)	15 (15.46)	45 (29.22)	
FIGO stage, *n*(%)				**0.004**
IB1–IIA2	64 (25.50)	34 (35.05)	30 (19.48)	
IIB–IIIB	128 (51.00)	49 (50.52)	79 (51.30)	
IIIC–IV	59 (23.51)	14 (14.43)	45 (29.22)	
Vaginal invasion, *n*(%)				**0.004**
≤1/2	184 (73.31)	81 (83.51)	103 (66.88)	
>1/2	67 (26.69)	16 (16.49)	51 (33.12)	
Surgery, *n*(%)				**0.001**
No	188 (74.90)	62 (63.92)	126 (81.82)	
Yes	63 (25.10)	35 (36.08)	28 (18.18)	
Para-aortic extension field, *n*(%)				0.308
No	224 (89.24)	89 (91.75)	135 (87.66)	
Yes	27 (10.76)	8 (8.25)	19 (12.34)	
Weekly chemotherapy, *n*(%)				**0.008**
No	76 (30.28)	20 (20.62)	56 (36.36)	
Yes	175 (69.72)	77 (79.38)	98 (63.64)	
Adjuvant chemotherapy, *n*(%)				0.203
<3 times	191 (76.10)	78 (80.41)	113 (73.38)	
≥3 times	60 (23.90)	19 (19.59)	41 (26.62)	

Bold values represent *p* < 0.05.

### 3.2 Dose-volume comparison of the intestine between the two groups

The dosimetric analysis demonstrated significant disparities in rectal irradiation parameters between the two cohorts (all *p* < 0.05; Table 2). Specifically, patients with grade ≥2 ARE exhibited markedly elevated rectal doses compared to the grade <2 group. The maximum rectal dose (Dmax_R) in the grade ≥2 cohort reached a median of 51.48 Gy (IQR: 48.85 ~ 53.11 Gy), exceeding the grade <2 group (median: 49.01 Gy, IQR: 48.24 ~ 52.35 Gy) by 2.47 Gy. Similarly, the mean rectal dose (Dmean_R) was significantly higher in the grade ≥2 group (median: 45.18 Gy, IQR: 43.41 ~ 46.43 Gy) compared to the grade <2 group (median: 43.47 Gy, IQR: 42.26 ~ 45.06 Gy), with a difference of 1.71 Gy. Volumetric parameters including V40_R and V50_R further emphasized this trend. These findings underscore the dose-dependent relationship between cumulative rectal irradiation and ARE severity. Notably, no significant differences were observed in small intestine dosimetric parameters (Dmax_SI, Dmean_SI, V30_SI, V35_SI, V40_SI; all *p* > 0.05; Table 2).

**Table 2:** Dose-volume comparison of the intestine between the two groups.

Variable	Total cases (*n* = 251)	Grade <2 ARE group (*n* = 97)	Grade ≥2 ARE group (*n* = 154)	*p*
Dmax_R (Gy)	51.08 (48.39, 52.88)	49.01 (48.24, 52.35)	51.48 (48.85, 53.11)	**0.002**
Dmean_R (Gy)	44.26 (42.68, 46.15)	43.47 (42.26, 45.06)	45.18 (43.41, 46.43)	**<0.001**
V40_R (%)	85.65 (74.74, 93.08)	81.04 (69.94, 88.31)	88.65 (79.03, 95.03)	**<0.001**
V45_R (%)	52.38 (41.20, 64.50)	51.05 (39.41, 58.99)	54.35 (43.05, 66.96)	0.202
V50_R (%)	5.39 (0.00, 19.80)	4.83 (0.00, 16.35)	9.04 (0.00, 21.64)	**0.002**
Dmax_SI (Gy)	47.31 (45.49, 55.47)	47.17 (45.47, 55.02)	47.21 (45.53, 56.12)	0.813
Dmean_SI (Gy)	16.75 (15.11, 18.74)	16.61 (15.43, 18.85)	16.53 (14.93, 18.99)	0.242
V30_SI (%)	18.71 (17.20, 25.78)	19.58 (17.52, 25.91)	18.48 (17.20, 25.65)	0.470
V35_SI (%)	10.30 (7.35, 20.83)	10.97 (7.69, 20.83)	10.10 (6.71, 21.34)	0.735
V40_SI (%)	4.73 (2.83, 15.53)	4.21 (2.27, 15.77)	4.90 (2.87, 16.46)	0.925

Bold values represent *p* < 0.05.

### 3.3 Multivariate logistic regression analysis for grade ≥2 ARE in older adult CC patients

Multivariate logistic regression analysis identified five independent predictors of grade ≥2 ARE in older adult CC patients undergoing radiotherapy (all *p* < 0.05; Table 3). Age demonstrated a modest yet significant association. Patients aged 70 years and older have a risk of ARE that is 1.88 times higher than those aged between 60 and 69 years (OR = 1.881, 95%CI: 1.015–3.484). Comorbid conditions showed stronger effects: hypertension quadrupled the risk (OR = 4.577, 95%CI: 2.402–8.720), while diabetes showed the highest predictive value (OR = 5.503, 95%CI: 2.206–13.726). Dosimetrically, every 1Gy increment in mean rectal dose (Dmean_R) elevated ARE risk by 30.9% (OR = 1.309, 95%CI: 1.155–1.483). As an emerging biomarker, LAR shows that for each unit increase in its value, there is an associated 87.2% increase in risk (OR = 1.872, 95%CI: 1.381–2.538).

**Table 3:** Multivariate logistic regression analysis for grade ≥2 ARE in older adult CC patients undergoing radiotherapy.

Variable	β	S. E	Wald χ^2^	*p*	OR (95%CI)
Age (y)					
60–69					1.000
≥70	0.632	0.315	2.008	**0.045**	1.881 (1.015–3.484)
Hypertension					
No					1.000
Yes	1.521	0.329	4.624	**<0.001**	4.577 (2.402–8.720)
Diabetes					
No					1.000
Yes	1.705	0.466	3.657	**<0.001**	5.503 (2.206–13.726)
Dmean_R (Gy)	0.269	0.064	4.226	**<0.001**	1.309 (1.155–1.483)
LAR	0.627	0.155	4.037	**<0.001**	1.872 (1.381–2.538)

Bold values represent *p* < 0.05.

### 3.4 Predictive ability of various factors for grade ≥2 ARE evaluated by ROC curve

**Figure 2:**
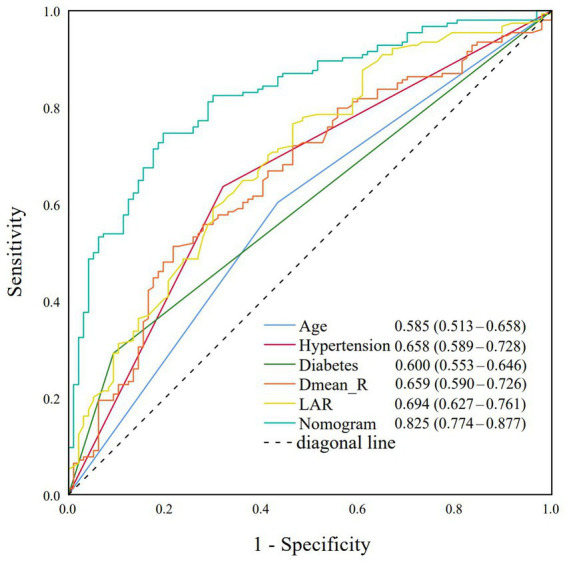
Receiver operating characteristic (ROC) curves for age, hypertension, diabetes, Dmean_R, LAR, and nomogram model.

Based on the multivariate logistic regression analysis, the predictive performance of individual risk factors and the nomogram model for grade ≥2 ARE was evaluated using receiver operating characteristic (ROC) curve analysis. The nomogram demonstrated superior discriminative ability with an area under the curve (AUC) of 0.825 (95% CI: 0.774–0.877), significantly superior to all individual parameters. Among single predictors, the LAR showed the highest AUC value (0.694, 95% CI: 0.627–0.761), followed by hypertension (AUC = 0.658, 95% CI: 0.589–0.728) and Dmean_R (AUC = 0.659, 95% CI: 0.590–0.726). Diabetes (AUC = 0.600, 95% CI: 0.553–0.646) and Age (AUC = 0.585, 95% CI: 0.513–0.658) showed more modest predictive capacities. The Youden index of each indicator was calculated separately to find the cut-off value. Among them, the diagnostic efficiency was the highest when Dmean_R was 45.24Gy and when LAR was 4.30 (Figure 2).

### 3.5 Forest plot of hazard ratios for grade ≥2 ARE by age categories

**Figure 3:**
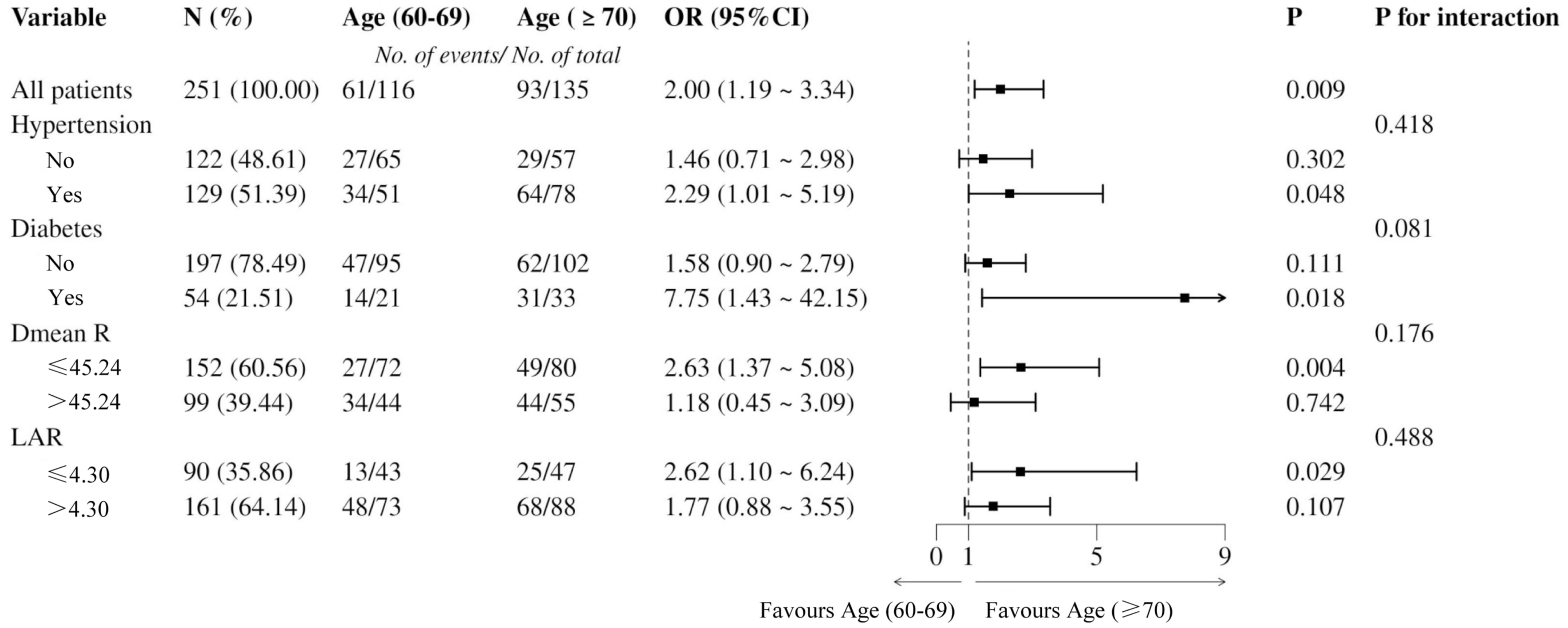
Forest plot of hazard ratios for grade ≥2 ARE by age categories.

Binary logistic regression was further performed to analyze ARE differences within age subgroups. The ≥70-year-old group had a significantly higher incidence of grade ≥2 ARE (93/135 vs. 61/116), with OR = 2.00 (95% CI: 1.19–3.34), *p* = 0.009 (Figure 3). The risk of grade ≥2 ARE was significantly increased among patients with hypertension, diabetes, and lower Dmean_R in the ≥70-year-old group (*p* < 0.05). No significant interactions were observed between subgroups, indicating that the effect of age on ARE risk was consistent across different subgroups (*p* > 0.05).

### 3.6 Construction and validation of the nomogram

**Figure 4:**
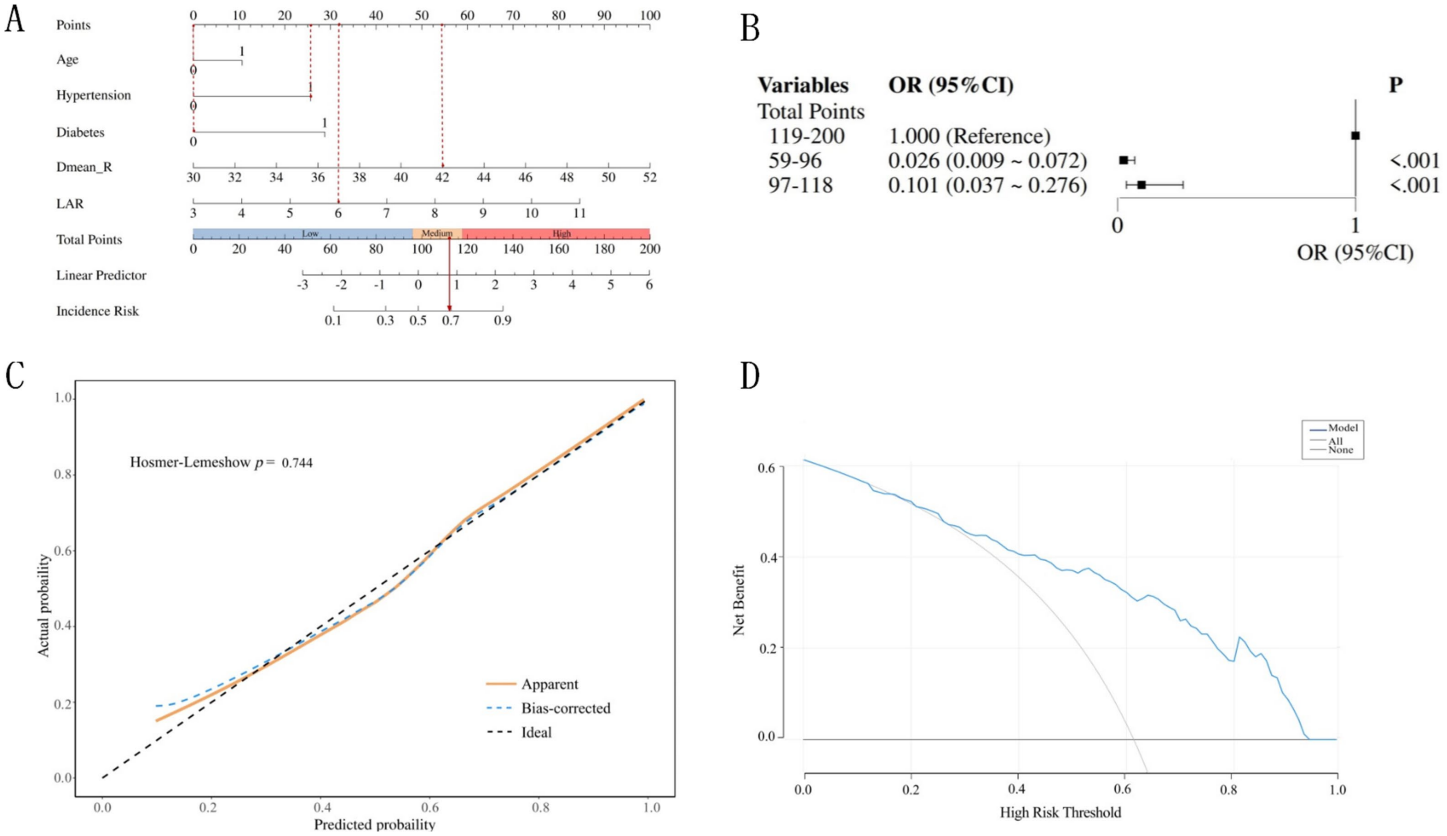
Construction and validation of the nomogram. **(A)** The nomogram model for prediction of Grade ≥2 ARE in older adult CC patients. **(B)** Association between the total points of the nomogram and Grade ≥2 ARE. **(C)** Calibration curves of the nomogram model predicting grade ≥2 ARE in older adult CC patients. **(D)** Decision curves of the nomogram model predicting grade ≥ 2 ARE in older adult CC patients.

The nomogram was constructed by integrating five independent predictors identified through multivariate logistic regression analysis (Figure 4A). Each variable—age, hypertension, diabetes, Dmean_R, and LAR—was assigned a weighted point value on a standardized scale. The total risk score, derived from summing these individual scores, corresponds to the predicted probability of an older adult CC patient developing grade ≥2 ARE on the nomogram’s risk axis.

To illustrate, consider a 66-year-old CC patient who has hypertension and has received a mean rectal dose of 42 Gy, and a laboratory test result for LAR = 6 prior to radiation therapy. The corresponding scores for each predictive variable are 0 points (Age), 26 points (Hypertension), 0 points (Diabetes), 54 points (Dmean_R), and 32 points (LAR), respectively. The patient’s cumulative score is 112 points, which corresponds to a 69% probability of grade ≥2 ARE.

The total points for all patients were calculated based on the nomogram and divided into three subgroups by tertile. These groups were then incorporated into a logistic regression model, demonstrating statistically significant differences among the three subgroups (*p* < 0.05) (Figure 4B). The risk of ≥Grade 2 ARE increased with higher total scores. Patients with lower total points (59–96) had a significantly lower risk compared to those with higher points (119–200; OR = 0.026, 95%CI: 0.009–0.072).

The nomogram prediction model was internally validated using the bootstrap method with self-sampling 1,000 times. Calibration analysis using the Hosmer-Lemeshow test confirmed excellent agreement between predicted and observed outcomes (*χ*^2^ = 5.127, *p* = 0.744) (Figure 4C). As illustrated in Figure 3, the calibration curve closely aligned with the ideal reference line (slope = 1), indicating robust model reliability for individualized risk stratification in older adult CC patients.

Decision curve analysis (DCA) revealed good clinical applicability of the nomogram, with threshold probabilities ranging from 0.21 to 0.93 (Figure 4D). This suggests the model’s robustness in guiding clinical decisions, validating the model’s capacity to optimize clinical decision-making by balancing overtreatment and undertreatment risks in older adult CC patients. The model performs best at moderate risk thresholds (approximately between 0.2 and 0.6), where using the model for decision-making is most reasonable. It can help doctors avoid overtreatment and undertreatment, thereby improving the net benefit of treatment.

## 4 Discussion

In modern gynecological oncology radiotherapy, despite the implementation of advanced techniques such as intensity-modulated radiation therapy (IMRT) and volumetric modulated arc therapy (VMAT), which significantly reduce radiation doses to organs at risk (OARs) (10–12), the incidence of ARE remains high in clinical practice (35.5–75.0%) (13–15). This phenomenon is particularly prominent in older adult patients, whose unique physiological characteristics and underlying comorbidities lead to significantly reduced tolerance to radiotherapy and a markedly increased risk of ARE compared to younger patients (4). In contrast to previous studies, this article investigates the indicators for predicting the risk of RE in older adult patients by focusing on their specific risk factors.

Our study enrolled 251 older adult cervical cancer patients (age ≥60 years) who underwent radiotherapy or concurrent chemoradiotherapy. The results showed that the overall incidence of ARE was as high as 82%, with 61% of cases being grade ≥2. Compared to previous reports of ARE incidence (54–75%) and grade ≥2 ARE incidence (29–56%) (16–18), the toxicity rates in our cohort were significantly higher. Multivariate analysis confirmed that age was an independent risk factor for grade ≥2 ARE. Notably, a clear age gradient effect was observed even within the older adult patient population. The relationship between age and radiotherapy toxicity remains controversial (19). However, the results of this study support the use of age as an important reference indicator for assessing treatment risks and determining radiotherapy doses in clinical practice.

Hypertension and diabetes, common comorbidities in older adult patients, were identified as independent risk factors for grade ≥2 ARE in this study. The underlying pathological mechanisms may involve reduced gut microbiota diversity, abundance, and gene counts in hypertensive patients compared to healthy individuals, with a decrease in beneficial bacteria and an increase in pathogenic microorganisms (20). Dysbiosis of the gut microbiota can lead to intestinal endothelial dysfunction, promote vascular sclerosis, and impair local tissue perfusion, further exacerbating tissue ischemia (21). This delays the repair of radiation-induced mucosal damage, thereby increasing the risk of severe RE. In diabetic patients, abnormal glucose metabolism has been shown to correlate positively with inflammatory responses (22). This synergizes with the non-specific inflammation triggered by radiation-induced intestinal injury, exacerbating oxidative stress and impairing tissue repair, creating conditions conducive to RE (23). Additionally, diabetic microangiopathy increases the risk of post-radiation intestinal damage (24). These pathological changes collectively make patients with hypertension and diabetes more sensitive to radiation damage, significantly increasing the risk of ARE.

In cervical cancer radiotherapy, the rectum and small intestine are the primary dose-limiting organs. When the cumulative dose of pelvic external irradiation exceeds 45–50 Gy, the risk of acute and late intestinal toxicity increases significantly (25). Therefore, strict control of rectal dose and volume in radiotherapy planning is crucial for reducing gastrointestinal toxicity. Compared to 3D conformal radiotherapy, IMRT has been proven to significantly reduce the incidence of grade 2 ARE in patients with late-stage rectal cancer (26). The small intestine, being highly sensitive to radiation, exhibits a close correlation between radiation dose and the occurrence of ARE. Studies have shown that prone positioning during gynecological radiotherapy can effectively reduce small intestine exposure, as this position allows the small intestine and parts of the colon to naturally shift away from the target area (18). Additionally, Chen et al. found that bladder filling status is closely related to the volume of small intestine within the planning target volume (PTV) (27). However, there is no consensus on the relative importance of various predictive factors. Through systematic analysis of dosimetric parameters for the rectum and small intestine, our study first confirmed that mean rectal dose (Dmean_R) is an independent risk factor for grade ≥2 ARE in older adult cervical cancer patients, consistent with the findings of Huang et al. (28). Notably, our study found no significant differences in small intestine dosimetric parameters between the two groups, aligning with Ma et al.’s conclusions (29). However, this result contrasts with some literature, which may be attributed to differences in radiotherapy techniques, bladder filling status, and patient positioning across studies. Therefore, in optimizing radiotherapy plans for older adult cervical cancer patients, priority should be given to rectal dose control, particularly limiting the mean dose (Dmean_R). At the same time, despite the lack of statistical significance for small intestine dosimetric parameters in this study, given the heterogeneity among studies, attention should still be maintained on small intestine dose parameters in clinical practice.

This study systematically evaluated the predictive value of various serum biomarkers for grade ≥2 ARE, with the lactate dehydrogenase to albumin ratio (LAR) demonstrating the best predictive performance (AUC = 0.694). As a composite indicator of the ratio of LDH to albumin, LAR integrates two key indicators to reflect the patient’s radiation tolerance. LDH, a critical enzyme in anaerobic glycolysis, directly reflects the degree of cellular damage and local hypoxia. Specifically, reactive oxygen species (ROS) released by inflammatory cells exacerbate oxidative damage, and ROS further enhance the inflammatory response. These interactions promote cellular damage and cell membrane rupture, which subsequently leads to the release of LDH. Studies have shown that elevated serum LDH levels correlate positively with the severity of tissue inflammatory injury. Serum LDH and lactate are risk factors for mortality in patients with severe inflammatory diseases (30). Albumin, an essential nutritional marker, also possesses anti-inflammatory and microcirculation-stabilizing properties (31). Hypoalbuminemia exacerbates radiation-induced microvascular damage and inflammatory responses. Thus, LAR levels reflect systemic inflammatory status. When radiation causes intestinal injury, released inflammatory factors disrupt the gut microenvironment and inhibit repair processes. Existing research indicates that LAR is an independent risk factor for poor prognosis in patients with lower respiratory tract infections (32), severe infections (33), and non-small cell lung cancer (34). To date, the role of LAR as a potential biomarker for radiation enteritis has not been explored. Our results demonstrated that LAR exhibited superior predictive performance (AUC = 0.694), better than other indicators such as diabetes (AUC = 0.600), although among the 54 diabetic patients, 45 exhibited higher-grade ARE. This may be because diabetes is a binary variable—while strongly associated with severe ARE (45/54 cases), it may not fully distinguish between the two groups. In contrast, LAR is a continuous biomarker that integrates LDH and albumin, enabling more sensitive risk stratification and capturing the inflammatory and nutritional status of all patients. This means that this simple and easily accessible indicator can effectively identify high-risk patients and provide important references for clinical decision-making.

There are many factors that influence ARE, and even experienced clinicians find it difficult to predict it early. Therefore, our nomogram, which includes clinical characteristics, dosimetric parameters, and novel biomarkers, was constructed based on five independent risk factors. We found that our predictive model demonstrated favorable predictive performance, with improved sensitivity and accuracy compared to individual predictors. Meanwhile, the calibration curve and decision curve analysis (DCA) indicated that the nomogram has good clinical applicability, thus providing more accurate risk stratification for older adult patients and avoiding overtreatment or undertreatment.

However, our study has several limitations. First, as a single-center retrospective study, the model was only internally validated within the enrolled cohort. Second, we only focused on the most clinically significant and common parameters. The limited sample size may introduce selection bias and affect the study’s reliability. Future research with larger sample sizes and external validation are warranted to further verify the model’s performance and enhance its generalizability.

## 5 Conclusion

In conclusion, age, hypertension, diabetes, Dmean_R, and LAR are independent risk factors for grade ≥2 ARE in older adult cervical cancer patients who received radiotherapy. A nomogram prediction model was established based on these factors. The developed nomogram integrates routine clinical indicators, balancing predictive accuracy and practicality, and serves as a valuable tool for improving radiotherapy safety in this vulnerable population.

## Data Availability

The original contributions presented in the study are included in the article/supplementary material, further inquiries can be directed to the corresponding authors.
